# ADHD genetic burden associates with older epigenetic age: mediating roles of education, behavioral and sociodemographic factors among older adults

**DOI:** 10.1186/s13148-023-01484-y

**Published:** 2023-04-26

**Authors:** Thalida E. Arpawong, Eric T. Klopack, Jung Ki Kim, Eileen M. Crimmins

**Affiliations:** Leonard Davis School of Gerontology, University of Southern California, Los Angeles, CA USA

**Keywords:** Attention deficit hyperactivity disorder, Neurodevelopment, Lifespan health, Epigenetics, Aging, DNA methylation, Epigenetic age

## Abstract

**Background:**

Shortened lifespans are associated with having Attention Deficit Hyperactivity Disorder (ADHD), which is likely mediated by related behavioral and sociodemographic factors that are also associated with accelerated physiological aging. Such factors include exhibiting more depressive symptoms, more cigarette smoking, higher body mass index, lower educational attainment, lower income in adulthood, and more challenges with cognitive processes compared to the general population. A higher polygenic score for ADHD (ADHD-PGS) is associated with having more characteristic features of ADHD. The degree to which (1) the ADHD-PGS associates with an epigenetic biomarker developed to predict accelerated aging and earlier mortality is unknown, as are whether (2) an association would be mediated by behavioral and sociodemographic correlates of ADHD, or (3) an association would be mediated first by educational attainment, then by behavioral and sociodemographic correlates. We evaluated these relationships in a population-based sample from the US Health and Retirement Study, among *N* = 2311 adults age 50 and older, of European-ancestry, with blood-based epigenetic and genetic data. The ADHD-PGS was calculated from a prior genomewide meta-analysis. Epigenome-wide DNA methylation levels that index biological aging and earlier age of mortality were quantified by a blood-based biomarker called GrimAge. We used a structural equation modeling approach to test associations with single and multi-mediation effects of behavioral and contextual indicators on GrimAge, adjusted for covariates.

**Results:**

The ADHD-PGS was significantly and directly associated with GrimAge when adjusting for covariates. In single mediation models, the effect of the ADHD-PGS on GrimAge was partially mediated via smoking, depressive symptoms, and education. In multi-mediation models, the effect of the ADHD-PGS on GrimAge was mediated first through education, then smoking, depressive symptoms, BMI, and income.

**Conclusions:**

Findings have implications for geroscience research in elucidating lifecourse pathways through which ADHD genetic burden and symptoms can alter risks for accelerated aging and shortened lifespans, when indexed by an epigenetic biomarker. More education appears to play a central role in attenuating negative effects on epigenetic aging from behavioral and sociodemographic risk factors related to ADHD. We discuss implications for the potential behavioral and sociodemographic mediators that may attenuate negative biological system effects.

**Supplementary Information:**

The online version contains supplementary material available at 10.1186/s13148-023-01484-y.

## Background

Biological aging has recently been measured through epigenetic age scores, which quantify the degree of methylation marks across one’s epigenome. Higher scores for one score in particular, called *GrimAge*, show associations with having more disability, disease co-morbidities, and shorter survival time compared to the same chronologically-aged peers [1]. Also associated with all of these factors is having health and behavioral symptoms associated with Attention Deficit Hyperactivity Disorder (ADHD) [2–8]. ADHD is characterized by challenges of sustained attention and concentration, increased impulsivity, affective disorders, and emotional dysregulation [8–12]. In turn, these challenges associate with downstream social and academic problems among adolescents, as well as less healthy behaviors such as poor dietary regulation, maladaptive coping abilities, and more substance use throughout life [7, 13–17]. Individuals with a higher genetic burden for ADHD, indexed through an ADHD polygenic score (ADHD-PGS), are also at higher risk for the same co-occurring behavioral factors and co-morbidities [3, 18]. Assessing relationships between ADHD genetic burden, via the ADHD-PGS, and epigenetic *GrimAge* can improve our understanding of pathways through which risk for earlier mortality is elevated for those with ADHD. This may be useful for clinical and geroscience researchers [19, 20] in assessing the utility of this epigenetic biomarker among an understudied group of older individuals at elevated risk for shorter health- and lifespans due to features of ADHD.

### ADHD polygenic score to represent elevated risk for ADHD characteristics

In larger population-based studies, it is often impossible to obtain clinical assessments for diagnoses of ADHD on respondents [21, 22]. Additionally, high heterogeneity exists in what leads to a confirmed diagnosis in adults [12, 23–25], for example, changes in criteria [26] defined by the Diagnostic and Statistical Manual of Mental Disorders (DSM), inconsistent informant ratings used in symptom reports [27], and different types of behavioral challenges, or psychiatric co-morbidities present at the time one is evaluated for a diagnosis [28, 29]. There are also varying degrees of symptoms that occur at different times across the lifespan [30, 31], with no clear clinical cut-points for diagnosis [12]

There are several genetic variants that have been consistently associated with ADHD, yet the underlying molecular genetics are not fully understood [12, 27]. Overall, the literature indicates that genetic contributions to ADHD are consistently high, with twin and family studies estimating heritability for ADHD to be 74% throughout life [27, 32–35]. Polygenic scores (PGS) are calculated by summing effects of multiple genetic markers across the genome [27]. The ADHD-PGS has shown dose-dependent relationships whereby increasing deciles of the score are associated with increasingly greater odds for an ADHD diagnosis (average OR = 1.56, 95% confidence interval: 1.53–1.60) [18, 36]. There is no established diagnostic threshold for the ADHD-PGS [18, 37]. Yet, polygenic scores for ADHD can be calculated to represent a continuous range in which individuals with higher scores are likely to meet criteria for a clinical diagnosis, or exhibit more characteristics typical of an ADHD diagnosis [18, 38].

### Features of ADHD and earlier age of mortality

Individuals with any of the behavioral and sociodemographic characteristics commonly associated with ADHD carry a twofold greater risk for earlier death in childhood compared to the general population [5]. A recent meta-analysis partially attributed this to unnatural causes (e.g., accidents, unintentional injuries, suicide) rather than to natural causes (e.g., neurologic, respiratory problems, cancer), although specific behavioral mediators were not assessed. Another study estimated that due to 14 adverse sociodemographic, health and behavioral mediators, those with ADHD showed a 12.7-year reduction in healthy life expectancy before age 27 [7]. Mediating factors included age, gender, weight and height, educational attainment, income, smoking status, frequency of exercise, alcohol use, diet, typical sleep duration, subjective health state, presence of Type 2 diabetes, and risky driving. Even those who no longer met the diagnostic criteria for ADHD at follow-up retained a 9.6-year reduction in healthy life expectancy based on these risks. Other large-scale studies similarly find that cause-specific mortality for those with ADHD is related to psychiatric co-morbidities [4, 39].

When evaluating health outcomes with aging, including cognitive outcomes, mental health and age at mortality, one factor that is consistently protective against worse outcomes is more educational attainment [40–42]. Individuals with more features of ADHD tend to pursue less education because of behavioral issues that interfere with learning in traditional classrooms, learning challenges in core subjects, and low availability of targeted academic support [43–45]. Although, with the critical role education plays for multiple aging-related and health outcomes, examining how education can mitigate the adverse circumstances associated with ADHD, and attenuate poorer outcomes such as shortened lifespans is important. Also clinically useful is examining how the effects of these factors altogether are reflected in a measurable biomarker of accelerated biological aging and earlier mortality.

### Epigenetic GrimAge and ADHD genetic burden

Use of the epigenome has become a promising method for indexing accelerated biological aging when the degree of methylation marks is quantified into indices previously shown to predict lifespan, healthspan or physiological dysfunction [1, 46–51]. Only two studies to date have examined how the ADHD-PGS relates to methylation levels [52, 53]. One study showed no relationship between the ADHD-PGS and the first-generation Horvath clock [54] among a young sample, ages 7 to 12 years [52]. In contrast, the other study found that among adults, a higher ADHD-PGS was associated with lower global methylation levels among ADHD cases, but not among controls [53]. Additionally, cases showed significantly lower global methylation levels overall and greater prevalence of externalizing conditions, including nicotine use, alcohol use, substance use, oppositional defiance, major depression, generalized anxiety, and bipolar disorder [53]. Relatedly, lower global methylation levels have also been associated with older age, and with greater physiological decline [55, 56]. Thus, methylation levels appear to reflect some degree of difference in age-related impairment including for those with greater ADHD genetic burden or a diagnosis, although such associations may not be captured by all epigenetic clocks.

Methylation at specific regions has also been consistently associated with multiple environmental factors and behaviors, for example, environmental pollution [57, 58], tobacco smoke [59, 60], obesity [61], and adverse childhood stress experiences [62]. Prior research has shown that regions encompassed in the *GrimAge* clock are strongly associated with earlier physiological and functional declines (e.g., cancers, chronic disease, immune system dysfunction, endocrine declines). *GrimAge* has demonstrated stronger relationships with all-cause mortality [48] and aging-related clinical phenotypes, including functional ability, cognition, and frailty [63]. Recently, *GrimAge* has been shown to be a stronger predictor of mortality than the Horvath clock, with effects that are independent of genetic influences [64]. Thus, as a second-generation clock, *GrimAge* may be better suited for indexing accelerated aging and risk for earlier mortality related to specific health risks related with ADHD.

### The current study

**Fig. 1:**
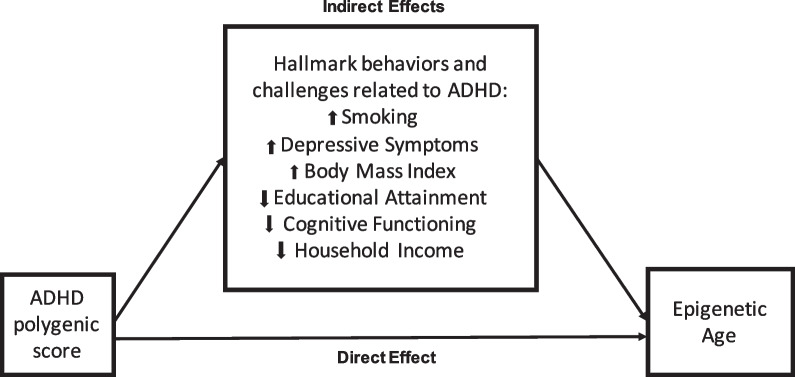
Proposed mediating pathways for the relationships between an Attention Deficit Hyperactivity Disorder (ADHD) polygenic score and epigenetic aging, through the hallmark behaviors and challenges related to ADHD

Given the consistency of research on the relationships between features of ADHD and risk for earlier mortality [3, 5, 7, 8], it is likely that the association between higher ADHD-PGSs and older biological age is mediated by some of the same adverse health behaviors and sociodemographic correlates that result in reduced life expectancy for those with ADHD [7]. These are shown in Fig. 1. First, we test the hypothesis that an ADHD-PGS associates with the epigenetic age marker for shortened lifespan, *GrimAge*. Second, we evaluate the hypothesis that health behaviors and sociodemographic correlates believed to be on the etiological pathway from ADHD to reduced life expectancy are similarly on the etiological pathway from the ADHD-PGS to older epigenetic age. Third, we test the hypothesis that the effects of the ADHD-PGS on *GrimAge* are initially mediated through educational attainment, then through the other factors. Findings from this study have the potential to identify life-course pathways through which the ADHD-PGS and related symptoms associate with earlier mortality and could identify potential targets that attenuate biological system effects.

## Methods

### Participants

The sample was drawn from the *Health and Retirement Study* (HRS), a nationally representative sample of households of older Americans (aged 51 + years) in the USA. Data collection for the HRS began in 1992 and continues to be collected bianually [65]. The HRS is funded by the National Institute on Aging and is administered by the University of Michigan, where there is Institutional Review Board approval for the study. Participants consent to be interviewed biennially on a broad range of economic, psychological, physical, and biological health measures [66]. For the present study, we included all participants who were interviewed in 2016 and provided a venous blood sample with which to characterize DNA methylation levels [67], who participated in an enhanced face-to-face interview (in 2006, 2008, 2010, 2012) to provide a saliva sample with which to characterize individual genotypes to calculate ADHD genetic scores and provided responses to surveys at baseline or subsequent waves (through 2016) to calculate cognitive functioning, BMI, lifetime smoking dosage, depression history, and educational attainment. In total, the HRS calculated polygenic scores on 12,900 respondents of European ancestry who provided genetic data through 2012. The total sample that provided DNA methylation data in 2016 included 4018 individuals, in which 2344 were overlapping with the genetic subsample. Of those, *n* = 2311 had complete data on all other variables and created the analytic sample.

### Measures

#### Epigenetic biological age score

DNA methylation was assessed using the Infinium Methylation EPIC BeadChip (Illumina, Inc., San Diego, CA). Preprocessing and quality control were completed using the *minfi* package in R, with removal of suboptimal samples or sex mismatched samples thereby yielding 836,660 methylation probes. Epigenetic age as measured by *GrimAge* was calculated by HRS [68]. As described in a prior publication [1], the clock was developed using a two-stage process in which developers first identified DNA methylation surrogates for physiological risk and stress factors (i.e., plasma proteins, growth differentiation factors) and smoking pack-years. Second, time to death due to all-cause mortality was regressed in elastic net Cox regression models on the DNA methylation surrogates and estimator of smoking pack-years to identify 1030 CpG sites that were then used to calculate a composite epigenetic score intended to estimate epigenetic age and predict morality risk. The slope and intercept terms of the GrimAge score calculation were then chosen to match the mean and variance of chronological age in the training data so that GrimAge can be interpreted in units of years. The score has demonstrated high and replicative properties for predicting time to death and onset of coronary heart disease, cancer and other age-related conditions [1, 47, 63].

#### Polygenic score for ADHD (ADHD-PGS)

Genotyping was performed by the National Institutes of Health (NIH) Center for Inherited Disease Research (CIDR; Johns Hopkins University, Baltimore, MD) using the Illumina Human Omni2.5-Quad BeadChip (Illumina, San Diego, CA), with coverage of nearly 2.5 million single nucleotide polymorphisms (SNPs). All quality control checks and imputation procedures were implemented by the HRS with details provided elsewhere [69, 70]. Related individuals were removed after estimation of kinship coefficients. The genotyped data were then imputed to the 1000 Genomes reference panel, phase 3, version 5 using Minimac, with phasing performed using SHAPEIT2. From the genotyping data, HRS calculates and distributes polygenic scores for several traits [71]. The PGS for ADHD was created from publicly available results of a meta-analysis of genomewide association scans across 12 cohorts (*n* = 55,374 from 20,183 cases and 35,191 controls) [18], with the discovery cohort from the Integrative Psychiatric Research (iPSYCH) initiative and remaining 11 cohorts from the Psychiatric Genomics Consortium (PGC). ADHD-PGSs were calculated by weighted sums of SNPs in which the weights were generated by the meta-analysis. The PGS includes 12 genetic variants that surpassed genome-wide significance in the discovery sample, 10 of which validated in the replication sample. In total, the ADHD-PGS includes 1,043,408 SNPs that were in common between the HRS imputed data and the meta-analysis. Scores were then standardized within the HRS cohort to a mean of 0 and standard deviation of 1. Because genetic risk scores for ADHD were derived from a meta-analysis of primarily European ancestry individuals and evidence that polygenic indices are sensitive to genetic substructure and ancestry differences [72, 73], this study includes only the European ancestry sample.

#### Health behaviors and sociodemographic covariates

For cigarette smoking, the National Cancer Institute definition of smoking pack-years was calculated as an indicator of lifetime smoking, as a multiplicand of smoking duration in years and average cigarettes per day, then divided by 20. Variables used for coding pack-years included age of start, age of stop, and cigarettes smoked per day extracted from HRS 1992–2016 core wave files. In the case of a missing start age, the mean start age was used, calculated for each gender and 10-year birth cohort (e.g., < 1920, 1920–29, 1930–39, etc.). In the case of missing cigarettes smoked per day, the mean was used, calculated for each gender, 10-year birth cohort, and stratified by current/former smoking status. In the case of missing stop age, the mean stop age was used, calculated for each gender and 10-year birth cohort for former smokers.

Depressive symptoms were assessed using a modified version of the well-known Center for Epidemiologic Studies-Depression (CES-D) inventory [74, 75] at baseline. The HRS uses 8 of the original items selected for their psychometric properties and assesses the continuum of depressive symptoms [76]. A continuous sum score of number of symptoms was used, which has demonstrated similar construct and external validity to those based on the original inventory [77, 78].

Body Mass Index (BMI), cognition, and income variables were coded from the HRS baseline wave for each respondent, or the subsequent wave to study entry if baseline data were missing. A continuous variable was used for BMI, calculated as kilograms of weight divided by height in meters squared (kg/m^2^). A continuous composite score for cognitive functioning was used, calculated as a sum score (range 0 to 27) with a higher score indicating fewer challenges with cognitive processes, including domains of episodic memory, working memory, sustained attention, and orientation [79]. Total income in adulthood was calculated as a sum of the income of respondent and their spouse if applicable. Because of the wide range of income, from $0 to over $2 M, we categorized it into quartiles with cut-off points at $16,800, $36,820 and $73,560.

Years of education attained were assessed as total number of years of school completed with a maximum of 17 years at the high end, indicating schooling beyond a college degree.

Other study covariates included self-identified gender (0 = female, 1 = male) that was verified genetically, chronological age in years, and the top six eigenvalues from principal component (PC) analysis of genotyped data to account for differences in population substructure [72, 80]. Six eigenvalues were selected for several reasons, including that prior analyses on ADHD-PGS and global DNA methylation indicated sufficient coverage using 5 PCs [53]; in preliminary regression models predicting GrimAge in our data including all 10 PCs, only PC1 was significantly associated with GrimAge (*p* = 0.02); recommendations in HRS quality control evaluations show sufficient coverage of population substructure problems when using 6–7 PCs [71]; and that we restricted the analysis to individuals of European ancestry to further reduce issues with population substructure.

### Statistical analyses

First, ordinary least-squares (OLS) regression models were constructed to evaluate the association between the ADHD-PGS and epigenetic age, adjusted for age, gender, and six ancestry principal components (PCs). Second, structural equation models (SEMs) were used to test indirect effects of the ADHD-PGS and epigenetic age through smoking pack-years, depressive symptoms, BMI, education, cognition and income with all mediators together. Covariances were added between all predictors. Third, multi-mediational SEMs were constructed to test whether there were indirect effects of the ADHD-PGS through education to influence all other mediators and GrimAge. All SEMs were adjusted for age and gender. Because in SEMs, PCs contributed 0 variance to the model, they were not included in SEMs. OLS models were constructed using SAS [81] and SEMs in Mplus [82].

We examined direct and indirect effects of each model and used common model fit indices to evaluate goodness of fit: root-mean square error of approximation (RMSEA), Tucker–Lewis Index (TLI), Comparative Fit Index (CFI), and Chi-square values to evaluate the best fitting model and p-value threshold of 0.05 for evaluating significance of path estimates. Generally, lower RMSEA values from 0.01, 0.05, 0.08 to 0.10 indicate the model has excellent, good, acceptable, and poor fit to the data, respectively [83, 84]; in contrast, the larger values for TLI and CLI at greater than 0.90 and 0.95 suggest good model fit [85, 86]. The direct effect in SEMs is defined as the effect of the ADHD-PGS (X) on GrimAge (Y) at the mean level of the mediator (M), and quantified by the path coefficient c. The indirect effect of the ADHD-PGS on GrimAge is quantified as the products of the path coefficients, a (from X to M) and b (from M to Y). Bias-corrected confidence intervals for indirect effects were calculated by bootstrapping with 5000 draws [87]. For path models with 80% power or greater to detect small effects in partial mediation, a single mediator model requires a sample size of 980 and multiple mediator model requires 920 [88]; thus, we were adequately powered with *n* = 2311 for this study.

Because chronological age showed high correlation with epigenetic age (*r* = 0.83), we ran all analyses adjusted for chronological age. Thus, we report all results using GrimAge scaled in units of years, and unstandardized coefficients to interpret effects in years of GrimAge.

## Results

### Sample characteristics

The analytic sample is comprised of 2311 adults who ranged between 50 and 98 in chronological age. As shown in Table 1, the epigenetic GrimAge score had a mean of 69.4 (SD = 8.7). The average epigenetic age for the sample was 2.2 years younger than the average chronological, or calendar age. The ADHD-PGS was standardized to a mean of 0 and standard deviation of 1, with scores ranging from −3.6 to 3.7. More than half of the sample was women, and on average individuals reported experiencing less than 1 depressive symptom in the last week, and mean lifetime pack-years among ever smokers of 27.5. Additionally, at least one-third of the sample fell into the obese range when defined as a BMI of 30 or greater. About 46.7% respondents completed at least 16 years of school, or the equivalent of a college degree, and the mean income was over $80,000.

**Table 1:** Sample characteristics for participants from the US Health and Retirement Study

Variable	Mean (SD) or *n* (%)
Epigenetic age in 2016: GrimAge (years)	69.4 (8.7)
Chronological age in 2016 (years)	71.6 (9.6)
ADHD polygenic score	0.0 (1.0)
Gender	
Male	996 (43.6%)
Female	1289 (56.4%)
Depressive symptoms	1.0 (1.6)
Smoking (lifetime pack years)	27.5 (26.2)
Body Mass Index (BMI) (kg/m^2^)	27.8 (5.6)
Cognition score	17.7 (3.6)
Education attained (years)	13.6 (2.4)
Income ($)	82,358.4 (100,592.4)

Range for depressive symptoms was 0 to 8, smoking pack-years were 0 to 156, BMI was 16.5 to 57.4, cognition score was 0 to 27, years of education was 0 to 17, income was 0 to 2.1 million

*SD* = standard deviation, *ADHD* = attention deficit hyperactivity disorder

### ADHD-PGS and epigenetic aging

As shown in Table 2, we found support for hypothesis 1. The ADHD-PGS, with each 1-SD increase, was significantly associated with almost half a year older GrimAge (*b* = 0.49, *p* < 0.0001). Each additional chronological year of age was associated with a 0.76 year older GrimAge (*b* = 0.76, *p* < 0.0001). Men had a 3.43 year higher GrimAge (*b* = 3.43, *p* < 0.0001) compared to women. All variables accounted for 74.85% of the variation in GrimAge, with 4.82% of the variation accounted for by gender, ADHD-PGS and ancestral principal components. The ADHD-PGS alone accounted for 0.29% of the variation in epigenetic age. Bivariate correlations among variables are provided in the Additional file 1: Table S1.

**Table 2:** Unstandardized coefficients from regression models for the direct association between the ADHD Polygenic Score (ADHD-PGS) and epigenetic aging, as indexed by the DNA methylation-based indicator of GrimAge

Parameter	Beta coefficient	SE	*p*-value
Intercept	74.79	0.30	< .0001
Age	0.76	0.01	< .0001
Gender: male	3.43	0.18	< .0001
ADHD-PGS	0.49	0.09	< .0001
Model *R*^2^			74.85%

Models are adjusted for ancestral principal components as covariates for population substructure

*SE* = standard error, *R*^2^ = Variance explained by the model

Because a prior study [52] found no association between the ADHD-PGS and the first-generation Horvath epigenetic clock often used to reflect biological aging, we tested the association in our sample to compare potential differences. We also found no association between the ADHD-PGS and the Horvath epigenetic clock (*p* = 0.56; details provided in the Additional file 1: Table S2).

### Indirect effects through adverse behavioral and sociodemographic mediators

**Fig. 2:**
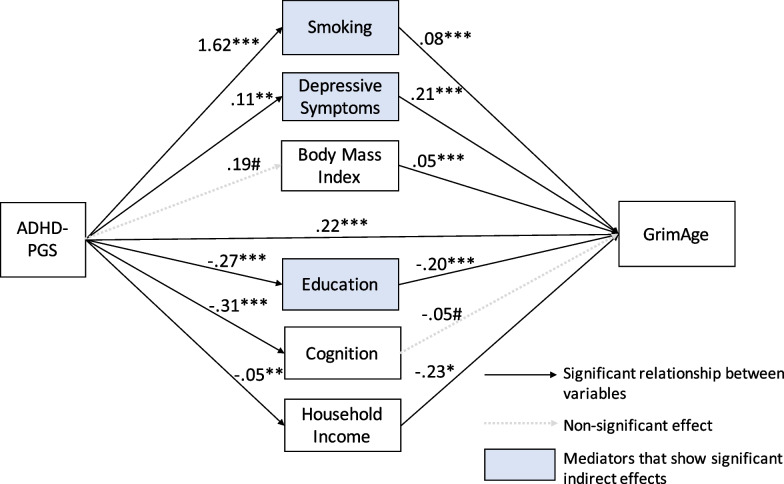
SEM results showing mediating pathways for the relationships between the ADHD polygenic score (ADHD-PGS) and GrimAge in later life. #*p* < .10, *p < .05, **p < .001, ***p < .0001

To test hypothesis 2, the model predicting GrimAge was constructed with all mediators in the model, as depicted in Fig. 2, and showed good fit (*χ*^2^(42) = 5159.42, *p* < 0.0001, RMSEA = 0.036, CFI = 0.998, TLI = 0.976). In this model, the ADHD-PGS directly associated with GrimAge, with each additional SD of the PGS associating with almost a quarter year older GrimAge (*b* = 0.22, *p* < 0.01). Smoking, depressive symptoms, and education mediated 51.7% of the effect of the ADHD-PGS on GrimAge as described below.

Indirect paths from the ADHD-PGS to GrimAge were significant through smoking (*b* = 0.13, *p* < 0.001), depressive symptoms (*b* = 0.02, *p* < 0.011), and education (*b* = 0.06, *p* < 0.0001), as shown by the blue shaded boxes in Fig. 2 and as listed in Table 3. Indirect paths were not significant through BMI (*b* = 0.009, *p* = 0.14), cognition (*b* = 0.01, *p* = 0.08), or income (*b* = 0.01, *p* = 0.07). Thus, mediational hypothesis 2 was partially supported.

**Table 3:** Unstandardized direct and indirect effects of the ADHD polygenic score (ADHD-PGS) on GrimAge for the single mediation model

Pathway	Path a	Path b	Path a*b: Indirect effects	Path c: Direct effect
	Beta (SE)	Beta (SE)	Beta (95% CI)	Beta (SE)
ADHD-PGS → GrimAge	–	–	–	0.222 (0.080)**
Via smoking	1.622 (0.482)***	0.077 (0.003)***	0.125 (0.064, 0.187) ***	
Via depressive symptoms	0.109 (0.033)**	0.214 (0.052)***	0.023 (0.009, 0.041)*	
Via body mass index	0.194 (0.115)#	0.046 (0.015)***	0.009 (0.000, 0.020)	
Via education	−0.269 (0.048)***	−0.204 (0.038)***	0.055 (0.032, 0.081)***	
Via cognition	−0.306 (0.073)***	−0.047 (0.024)#	0.014 (0.001, 0.030)#	
Via income	−0.052 (0.019)**	−0.231 (0.095)*	0.012 (0.002, 0.025)#	
Total indirect effect			0.238 (0.165, 0.315)***	

*SE* = standard error, *95% CI* = 95% confidence interval, estimated from a bias-corrected bootstrap procedure with 5000 draws

^#^*p* < .10; *p < .05; **p < .01; ***p < .001. Total effect on GrimAge: *β* = 0.460, SE = 0.090, *p* < .0001

All variables in the model accounted for 78.8% of the variation in GrimAge. This included direct effects, depicted in Fig. 2, in which the ADHD-PGS was positively associated with smoking pack years (*b* = 1.62, *p* < 0.001), which in turn, predicted older epigenetic age (*b* = 0.08, *p* < 0.0001). Similarly, higher ADHD-PGS was positively associated with depressive symptoms (*b* = 0.11, *p* < 0.001), which in turn, predicted older epigenetic age (*b* = 0.21, *p* < 0.0001). The ADHD-PGS was not related to BMI (*b* = 0.19, *p* = 0.09), but BMI significantly predicted older epigenetic age (*b* = 0.05, *p* < 0.001). Higher ADHD-PGS significantly and inversely associated with more educational attainment (*b* = −0.27, *p* < 0.0001) which, in turn, predicted younger GrimAge (*b* = −0.20, *p* < 0.0001). Higher ADHD-PGS significantly and inversely associated with higher cognitive scores (*b* = −0.31, *p* < 0.0001), but cognitive scores did not predict GrimAge (*b* = −0.05, *p* = 0.07). Lastly, higher ADHD-PGS significantly and inversely associated with higher income (*b* = -0.05, *p* < 0.01), which, in turn, significantly predicted younger GrimAge (*b* = −0.23, *p* < 0.05). Additional paths added for model fit and for covariates are provided in the Additional file 1 Table S3.

### Multi-mediation effects through education and other mediators

**Fig. 3:**
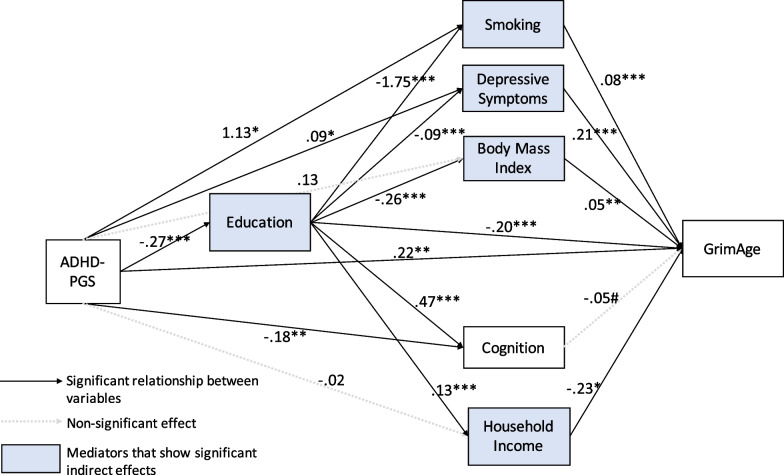
SEM results showing multiple mediation pathways for the relationships between the ADHD polygenic score (ADHD-PGS) and GrimAge in later life. #*p* < .10, *p < .05, **p < .001, ***p < .0001

To test hypothesis 3, multi-mediational models were constructed, as shown in Fig. 3. The model fit was good (*χ*^2^(42) = 5159.42, *p* < 0.0001, RMSEA = 0.030, CFI = 0.998, TLI = 0.983) with a similar or slightly better fit than the single mediation model as indicated by the RMSEA and TLI. In this model, as shown in Table 4, the association between ADHD-PGS and GrimAge was the same as for the single mediation model (*b* = 0.22, *p* < 0.01) as was the percentage of effect being mediated, 51.6%. The effect of the ADHD-PGS through education to GrimAge was secondarily mediated through smoking (*b* = 0.04, *p* < 0.0001), depressive symptoms (*b* = 0.005, *p* < 0.01), BMI (*b* = 0.003, *p* < 0.05), and income (*b* = 0.008, *p* < 0.05), but not via cognition (*b* = 0.006, *p* = 0.09). Thus, hypothesis 3 was partially supported.

**Table 4:** Unstandardized direct and indirect effects of the ADHD polygenic score (ADHD-PGS) on GrimAge for the multi-mediation model

Predictor or mediator	Path a1: ADHD-PGS → mediator	Path a2: Education → mediator	Path b: Mediator → GrimAge	Path a1*b: Indirect effects: ADHD-PGS → mediator → GrimAge	Path a2*b: Indirect effects: education → mediator → GrimAge	Path a1–a2–b: Indirect effects: ADHD-PGS → education → mediator → GrimAge	Path c: Direct effects: on GrimAge
	Beta (SE)	Beta (SE)	Beta (SE)	Beta (SE)	Beta (95% CI)	Beta (95% CI)	Beta (SE)
ADHD-PGS							0.222 (0.080)**
Smoking	1.134 (0.477)*	− 1.745 (0.202)***	0.077 (0.003)***	0.087 (0.026, 0.150)*	− 0.134 (− 0.163, − 0.106)**	0.036 (0.024, 0.050)***	
Depressive symptoms	0.086 (0.033)*	− 0.087 (0.014)***	0.214 (0.052)***	0.018 (0.005, 0.035)*	− 0.019 (− 0.029, − 0.010)**	0.005 (0.002, 0.009)**	
Body Mass Index	0.125 (0.115)	− 0.258 (0.049)***	0.046 (0.015)***	0.006 (− 0.002, 0.016)	− 0.012 (− 0.019, − 0.005)**	0.003 (0.001, 0.005)*	
Education	− 0.269 (0.048)***			0.055 (0.032, 0.081)***			− 0.204 (0.038)***
Cognition	− 0.180 (0.069)**	0.470 (0.030)***	− 0.047 (0.024)#	0.008 (0.000, 0.019)	− 0.022 (− 0.042, − 0.002)#	0.006 (0.001, 0.012)#	
Income	− 0.018 (0.018)	0.133 (0.008)***	− 0.231 (0.095)#	0.004 (− 0.003, 0.012)	− 0.031 (− 0.052, − 0.010)*	0.008 (0.002, 0.015)*	
Total Indirect Effect for ADHD-PGS to GrimAge						0.237 (0.165, 0.313)***	
Total indirect effect for education to GrimAge					− 0.217 (− 0.257, − 0.178)***		

*SE* = standard error, *95% CI* = 95% confidence interval, estimated from a bias-corrected bootstrap procedure with 5000 draws

^#^*p* < .10; *p < .05; **p < .01; ***p < .001. Total effect from ADHD-PGS to GrimAge: *β* = 0.459, SE = 0.090, *p* < .0001. Total effect from Education to GrimAge: *β* = −0.421, SE = 0.038, *p* < .0001

The ADHD-PGS, mediators, and covariates explained 80.5% of the variation in GrimAge. Direct effects from the ADHD-PGS include that it inversely predicted education (*b* = −0.27, *p* < 0.0001) and cognition (*b* = −0.18, *p* < 0.01), and positively predicted depressive symptoms (*b* = 0.09, *p* < 0.05) and smoking (*b* = 1.13, *p* < 0.05), but did not predict BMI (*b* = 0.13, *p* = 0.26), or income (*b* = −0.02, *p* = 0.31). Significantly and directly related to older GrimAge were smoking (*b* = 0.08, *p* < 0.0001), depressive symptoms (*b* = 0.21, *p* < 0.0001), BMI (*b* = 0.05, *p* < 0.001), less education (*b* = −0.20, *p* < 0.0001), less income (*b* = −0.23, *p* < 0.05), but not cognition (*b* = −0.05, *p* = 0.07). Notably, there was a significant direct effect from education to GrimAge (*b* = −0.20, *p* < 0.0001); with each additional year of education attained, there was a fifth of a year decrease in GrimAge.

Overall, the total indirect effect of the ADHD-PGS on GrimAge was (*b* = 0.24, *p* < 0.0001) proportionally similar, but in opposite direction to the total indirect effect of education on GrimAge (*b* = −0.22, *p* < 0.0001). As shown in Table 4, the specific indirect effect from the ADHD-PGS to GrimAge was significant via education (*b* = 0.06, *p* < 0.0001), via smoking (*b* = 0.09, *p* < 0.05), and via depressive symptoms (*b* = −0.02, *p* < 0.05), whereas non-significant paths were via BMI (*b* = 0.006, *p* = 0.30), via cognition (*b* = 0.008, *p* = 0.15), and via income (*b* = 0.004, *p* = 0.39). As shown by blue shaded boxes in Fig. 3, the specific indirect effects from education to GrimAge were significant via smoking (*b* = −0.13, *p* < 0.0001), via depressive symptoms (*b* = −0.02, *p* < 0.01), via BMI (*b* = −0.01, *p* < 0.01), and via income (*b* = −0.03, *p* < 0.05), but not via cognition (*b* = −0.02, *p* = 0.07). Additional paths added for model fit and for covariates are provided in the Additional file 1: Table S4.

### Sensitivity analysis for education

Because it is also possible that an ADHD-PGS underlies cognitive challenges that would likely affect years of schooling pursued, we conducted analyses to assess whether the results found could be due to a differential effect of education among the lowest and highest quartiles of the ADHD polygenic score. We constructed linear regression models to include an interaction term (ADHD-PGS*education), adjusted for the ADHD-PGS, education and covariates, and did not find evidence for the interaction (*b* = −0.04, *p* = 0.371) or when including only those in the lowest and highest quartiles of the polygenic score.

## Discussion

In this study, we evaluated whether an epigenetic marker for accelerated biological aging and earlier mortality was associated with ADHD genetic burden, and whether the association was mediated by hallmark behavioral and sociodemographic factors related to ADHD. We evaluated these mechanisms in a population-based sample, that includes adults over age 50, in which a polygenic score for ADHD represents a continuum of risk for having characteristic features of ADHD. Older biological age was indexed by a blood-based epigenetic biomarker called GrimAge, which has been associated with more disease co-morbidities and all-cause mortality. We found that the relationships with the epigenetic biomarker are partially mediated through educational attainment, lifetime smoking pack-years, and depressive symptoms, but not BMI, cognitive function or household income. Furthermore, there was evidence for pathways of effect in which the ADHD-PGS associated with lower educational attainment, which in turn associated with more smoking, depressive symptoms, higher BMI, and lower adult income to significantly affect GrimAge. While a higher ADHD-PGS increased epigenetic age, more education mitigated that effect, partially through behavioral and sociodemographic mediators. This implies that more educational attainment can mitigate negative outcomes related to damaging health and sociodemographic circumstances related to ADHD. Additionally, this epigenetic biomarker can be useful for indexing the effects of ADHD genetic burden and behavioral correlates of ADHD on multi-morbidity and lifespan.

### ADHD genetic burden and the epigenetic score to index epigenetic aging

We found that ADHD genetic burden is associated with GrimAge directly, with some of the effect mediated through behavioral and sociodemographic correlates. The direct relationship is likely attributable to both overlapping genetic and environmental influences. The extent to which DNA methylation is under the control of genetics specific to ADHD is largely unknown [89]. An analysis of differentially methylated regions via an epigenome-wide association scan conducted on the ADHD-PGS has shown that the methylation levels of one significant and 12 suggestive sites were not likely to be under genetic control [52]. Given that prior research has shown that ADHD genetic burden is associated with lower overall global methylation levels among ADHD cases, but not controls; that cases had significantly greater prevalence of specific behaviors (including smoking, alcohol, and substance use, major depression, generalized anxiety, and others [53]); and our finding that effects of ADHD genetic burden on GrimAge were mediated via behavioral factors (educational attainment, lifetime smoking pack-years, and depressive symptoms), taken together, these implicate that DNA methylation levels encompassed by the GrimAge clock are responsive to behavioral correlates related to ADHD [64]. This is promising given that behavioral correlates are more modifiable than one’s genetic code. Because ADHD genetic burden was unrelated to the first-generation epigenetic clock in a prior study and in ours, further research aimed at evaluating the ADHD relationship with other epigenetic clocks or differentially methylated regions would be useful to gauge clinical utility of other epigenetic markers for reflecting accelerated aging due to these behaviors.

It is well-known that genetics are not a lone predictor of ADHD or its features because a multitude of environmental components likely contribute, including early life exposures and contexts involved, but etiological pathways are poorly understood [12]. Emerging evidence suggests that DNA methylation could be part of the etiological pathway to ADHD [89] although we do not investigate etiology to ADHD in this study. Thus, future studies that can assess how there may be dynamic relationships between changes in both DNA methylation and ADHD-related behaviors over time would be useful for understanding how epigenetic scores can serve as useful biomarkers to assess accelerated aging. Longitudinal studies are also needed to evaluate how epigenetic biomarkers can change with respect to behaviors and socioeconomic contexts related to ADHD because different epigenetic signals might arise with different degrees of symptom presentation across the lifecourse. While this study could not assess change in epigenetic signals, we show that one epigenetic clock can serve as an omnibus older-age biomarker of accelerated biological aging related to genetic liability for ADHD and the related behavioral or sociodemographic circumstances salient to reduced lifespans for those with ADHD.

### Behavioral and sociodemographic correlates for ADHD and older epigenetic age

Less smoking, fewer depressive symptoms, lower BMI, and higher adulthood socioeconomic status were important for mitigating faster epigenetic aging and risk for earlier mortality due to ADHD genetic burden. The public health burden of any one of these health conditions at any age is substantial [90, 91], yet individuals with ADHD have elevated likelihood for exhibiting all of these features and particularly are at 50 to 300% elevated risk for the mental health and substance use factors [92–94]. These links between ADHD and multiple adverse health mediators heighten the public health significance for individuals with higher ADHD genetic burden and thus, identifying useful clinical biomarkers of health could facilitate their use in preventive health care.

What is most striking from our findings is that there is a primary mediating role of educational attainment and thus, points to the potential role it can play in mitigating adverse behaviors, lifestyle factors, accelerated aging and earlier mortality. Our results indicate that greater educational attainment could nullify the direct effect of ADHD genetic burden on GrimAge. However, there are good reasons that individuals with ADHD and related features do not pursue higher education, stemming from learning and behavioral challenges and often the lack of educational supports. Educational outcomes have also been linked to neurocognitive functioning across multiple domains, in which there tend to be deficits or delays in development among those with ADHD [12, 95]. Thus, our findings prompt the question of whether pursuing more education assists with greater development of neurocognitive abilities in order to abate the secondary adverse behavioral and lifestyle factors leading to shortened lifespans and accelerated epigenetic aging. An answer to this question may make our finding regarding primary mediation of health variables on GrimAge, as an index of mortality risk, through education particularly important as a health and prevention target for those with high varying degrees of genetic burden for ADHD. Furthermore, it would be useful to conduct additional studies to evaluate potential mechanisms through these other mediators and whether the degree of variation in and prediction from ADHD genetic burden varies due to environmental factors, socioeconomic resources, and how these might modify the relationships with epigenetic age or mortality risk.

### Limitations

Despite the strength of this study with the indexing of epigenetic age in older adulthood and mediating sociodemographic and behavioral pathways, we acknowledge several limitations. This study represents a snapshot in older age, for those at different levels of polygenic indices for ADHD who have survived to older adulthood. The sample was older at the outset than those typically assessed for features of ADHD, so it is unknown how such symptoms may attenuate in adulthood. It is also unknown how behavioral variables, including medication use, may have changed throughout the lifecourse and what effects they would have in accounting for the ADHD-PGS to epigenetic age relationship.

A prior evaluation of age patterns has shown that log odds for mortality due to ADHD increase in the age ranges of 46–64 [6]. Thus, it is possible that those who had the highest risk for shortened lifespan due to higher ADHD genetic burden are not included in the study. However, we are studying effects in an age range when those who have survived have experienced cumulative systems damage that is still reflected in older epigenetic age. Also, epigenetic age in the analytic sample is younger than chronological age, which means that on average, there is a distribution of healthier individuals who were included in the present study. Hence, any effects we detect are likely attenuated.

Another limitation is not considering gene-by-environment effects when there is likely a dynamic interplay occurring between genetic regions encompassed within the polygenic score and sociodemographic or behavioral factors. There is also a limitation of temporality because DNA methylation changes may occur earlier in life, with prior evidence showing for example that early life stress experiences can alter methylation levels [96, 97]. These early changes in methylation signatures can contribute to the etiological manifestation of mediators being evaluated rather than the mediators temporally predicting the methylation signatures. Future studies with multiple time-points of methylation assessment are needed to evaluate this.

Additionally, we do not look at onset of co-morbidities, such as age of onset of disease, which can differentially affect mortality risk. All measures for this study were taken in middle to later adulthood, so it is unknown how behaviors from earlier life affect the relationship between a polygenic score for ADHD and epigenetic age. However, a strength of this study is assessing a marker of the biological clock and aging real-time in a naturally aging sample. Because the sample is drawn from a population-based study, recruitment was not based on disease status or genetic risk, and thus, results give us a representation of today’s middle to older aged adults across a range of both DNA methylation levels and ADHD genetic burden.

## Conclusions

For geroscience research, we expand on the current knowledge to show that this blood-based index of epigenetic age can be used to evaluate the degree to which risk for earlier mortality differs based on mediating factors related to ADHD genetic burden. Research to identify links between ADHD and epigenetics is in the very early stages [89] overall. We show that an epigenetic score could serve as a monitoring tool for degree of biological damage garnered from adverse behavioral and sociodemographic conditions for which those with greater ADHD genetic burden are at high risk. More work is also needed to unpack the potentially pivotal role educational attainment has for mitigating adverse life circumstances related to ADHD genetic burden that accelerate biological aging that is reflected in GrimAge. Furthermore, findings from this study emphasize the relevance of integrating the behavioral and epigenetic markers in this understudied group of older individuals at elevated risk for shorter health- and lifespans due to genetic liability for ADHD-related circumstances.

## Supplementary Information

**Additional file 1**. Supplementary Tables and Material.

## Data Availability

The data that support the findings of this study are available from the University of Michigan, to approved investigators. Investigators can apply for use here: https://hrs.isr.umich.edu/about.

## Abbreviations

ADHD: Attention Deficit Hyperactivity Disorder
ADHD-PGS: Attention Deficit Hyperactivity Disorder Polygenic Score
BMI: Body mass index
CES-D: Center for Epidemiologic Studies-Depression
CFI: Comparative Fit Index
CI: Confidence interval
CIDR: Center for Inherited Disease Research
DNA: Deoxyribonucleic acid
HRS: Health and Retirement Study
iPSYCH: Integrative Psychiatric Research
NIH: National Institutes of Health
PC: Principal component
PGC: Psychiatric Genomics Consortium
RMSEA: Root-Mean Square Error of Approximation
OLS: Ordinary least squares
SD: Standard deviation
SE: Standard error
SEM: Structural equation modeling
SNP: Single nucleotide polymorphism
TLI: Tucker Lewis Index
