# Supplementary material for: ADHD genetic burden associates with older epigenetic age: mediating roles of education, behavioral and sociodemographic factors among older adults
Source: Clin Epigenetics. 2023 Apr 26;15:67. doi: 10.1186/s13148-023-01484-y (PMC10131361; doi:10.1186/s13148-023-01484-y)
Supplement: Supplementary file 1 — Additional file 1. Supplementary Tables and Material. [file 13148_2023_1484_MOESM1_ESM.docx]

**ADHD genetic burden associates with older epigenetic age: Mediating roles of education, behavioral, and sociodemographic factors among older adults**

**Authors:** TE Arpawong, ET Klopack, JK Kim, EM Crimmins

**Supplemental Information**

**Table S1.** Bivariate Correlations Among Key Variables

|  | GrimAge | ADHD-PGS | Age | Gender | Depression  symptoms | Smoke | BMI | Cognitive Function | Education |
| --- | --- | --- | --- | --- | --- | --- | --- | --- | --- |
| Epigenetic age: GrimAge |  |  |  |  |  |  |  |  |  |
| ADHD-PGS | 0.06* |  |  |  |  |  |  |  |  |
| Age | 0.84*** | 0.02 |  |  |  |  |  |  |  |
| Gender: male | 0.20*** | -0.02 | 0.01 |  |  |  |  |  |  |
| Depression symptoms | -0.08*** | 0.06* | -0.16*** | -0.07** |  |  |  |  |  |
| Smoking pack-years | 0.31*** | 0.05* | 0.05*** | 0.17*** | 0.05* |  |  |  |  |
| Body Mass Index (BMI) | -0.12*** | 0.01 | -0.19*** | 0.08*** | 0.08*** | -0.03 |  |  |  |
| Cognitive Functioning | -0.10*** | -0.08*** | -0.04 | -0.15*** | -0.08*** | -0.07** | -0.02 |  |  |
| Education years | -0.23*** | -0.11*** | -0.14*** | 0.08*** | -0.10*** | -0.15*** | -0.06** | 0.32*** |  |
| Household Income | -0.27*** | -0.04 | -0.18*** | 0.02 | -0.05 | -0.09*** | -0.02 | 0.13*** | 0.26*** |

Notes: ADHD-PGS = Attention Deficit Hyperactivity Disorder Polygenic Score. *p<.05; **p<0.01; ***p<0.001

*Sensitivity Checks on Effects of Education*

**Methods**

*Polygenic score for Educational Attainment (Edu-PGS).* The polygenic score for educational attainment was based on a meta-analytic genomewide association scan on over 400,000 individuals (293,723 individuals in the discovery cohorts, and 11,349 in the replication cohort) conducted by the Science and Genetic Association Consortium (SSGAC) and described in a prior publication by Okbay et al [1]. For calculation of the polygenic score in HRS, new SNP weights were provided by the SSGAC to the HRS, excluding two samples that were in the original cohorts: 23andMe (due to data use restrictions) and the HRS. Details are provided in HRS documentation [2]. Briefly, 1,309,267 SNPs in the European ancestry individuals in HRS were used to calculate scores that were then standardized to a mean of 0 and standard deviation of 1.

**Results**

Because there is overlap in the polygenic score for ADHD and polygenic indices for educational attainment (r=-0.28, p<.0001), we constructed ordinary least-squares (OLS) regression models to test whether the relationships between epigenetic age and either ADHD-PGS or years of education attained was accounted for by the polygenic score for educational attainment (Edu-PGS). When adding the Edu-PGS, we found it was not significantly associated with epigenetic age (β=-0.18, SE=0.11, p=.082), and the effect of the ADHD-PGS (β=0.19, SE=0.10 p=.059) was attenuated whereas years of education (β=-0.35, SE=0.04, p<.0001) remained significantly associated.

*Association between ADHD-PGS and Horvath Epigenetic Clock*

**Methods**

*Horvath Epigenetic Clock.* This clock was calculated by HRS as described in the documentation[3], based on the 353 CpG DNA methylation sites with detailed methods on the derivation of these sites published previously[4].

**Results**

**Table S2.** Unstandardized Coefficients from Regression Models for the Direct Association between the ADHD Polygenic Score (ADHD-PGS) and Epigenetic Aging, as Indexed by the Horvath Epigenetic Clock

| **Parameter** | **beta coefficient** | **Std. Err.** | ***p*-Value** |
| --- | --- | --- | --- |
| Intercept | 69.43 | 0.45 | <.0001 |
| Age | 0.73 | 0.28 | <.0001 |
| Gender: male | 1.24 | 0.28 | <.0001 |
| ADHD-PGS | 0.08 | 0.14 | 0.559 |
| Model R^2^ |  |  | 53.64% |

Notes. Std. Err. = standard error. R^2^ = variance explained by the model. Models are adjusted for ancestral principal components as covariates to adjust for population substructure.

**Table S3.** SEM results for single-mediation for the relationship between the Attention Deficit Hyperactivity Disorder Polygenic Score (ADHD-PGS) and GrimAge

| **Outcome** | **Predictor** | **beta** | **Standard Error** | ***P*-Value** |
| --- | --- | --- | --- | --- |
| GrimAge | ADHD-PGS | 0.222 | 0.081 | 0.006 |
|  | Age | 0.743 | 0.009 | <0.0001 |
|  | Gender | -2.837 | 0.167 | <0.0001 |
|  | Smoking | 0.077 | 0.005 | <0.0001 |
|  | Depressive Symptoms | 0.214 | 0.054 | <0.0001 |
|  | Education | -0.204 | 0.039 | <0.0001 |
|  | Income | -0.231 | 0.096 | 0.016 |
|  | Cognition | -0.047 | 0.025 | 0.067 |
|  | BMI | 0.046 | 0.014 | 0.001 |
| Smoking | ADHD-PGS | 1.622 | 0.487 | 0.001 |
|  | Gender | -8.257 | 1.023 | <0.0001 |
| Depressive Symptoms | ADHD-PGS | 0.109 | 0.035 | 0.002 |
|  | Gender | 0.278 | 0.064 | <0.0001 |
|  | Age | -0.027 | 0.004 | <0.0001 |
| BMI | ADHD-PGS | 0.194 | 0.110 | 0.079 |
|  | Gender | -1.260 | 0.227 | <0.0001 |
|  | Age | -0.092 | 0.012 | <0.0001 |
| Education | ADHD-PGS | -0.269 | 0.048 | <0.0001 |
|  | Gender | -0.262 | 0.101 | 0.01 |
| Cognition | ADHD-PGS | -0.306 | 0.071 | <0.0001 |
|  | Age | -0.002 | 0.008 | 0.806 |
|  | Gender | 1.083 | 0.149 | <0.0001 |
| Income | ADHD-PGS | -0.052 | 0.019 | 0.005 |
|  | Age | -0.023 | 0.002 | <0.0001 |
|  | Gender | -0.138 | 0.038 | <0.0001 |

**Table S4.** SEM results for multiple-mediation for the relationship between the Attention Deficit Hyperactivity Disorder Polygenic Score (ADHD-PGS) and GrimAge

| **Outcome** | **Predictor** | **beta** | **Standard Error** | ***P*-Value** |
| --- | --- | --- | --- | --- |
| GrimAge | ADHD-PGS | 0.222 | 0.081 | 0.006 |
|  | Gender | -2.837 | 0.167 | <0.0001 |
|  | Age | 0.743 | 0.009 | <0.0001 |
|  | Smoking | 0.077 | 0.005 | <0.0001 |
|  | Depressive Symptoms | 0.214 | 0.054 | <0.0001 |
|  | Education | -0.204 | 0.039 | <0.0001 |
|  | Income | -0.231 | 0.096 | 0.016 |
|  | Cognition | -0.047 | 0.025 | 0.067 |
|  | BMI | 0.046 | 0.014 | 0.001 |
| Education | ADHD-PGS | -0.269 | 0.048 | <0.0001 |
|  | Gender | -0.263 | 0.101 | 0.01 |
| Smoking | ADHD-PGS | 1.134 | 0.484 | 0.019 |
|  | Education | -1.745 | 0.223 | <0.0001 |
|  | Gender | -8.650 | 1.006 | <0.0001 |
| Depressive Symptoms | ADHD-PGS | 0.086 | 0.034 | 0.012 |
|  | Education | -0.087 | 0.015 | <0.0001 |
|  | Gender | 0.227 | 0.063 | <0.0001 |
|  | Age | -0.030 | 0.004 | <0.0001 |
| BMI | ADHD-PGS | 0.125 | 0.110 | 0.255 |
|  | Education | -0.258 | 0.049 | <0.0001 |
|  | Gender | -1.406 | 0.225 | <0.0001 |
|  | Age | -0.102 | 0.012 | <0.0001 |
| Cognition | ADHD-PGS | -0.180 | 0.067 | 0.008 |
|  | Education | 0.470 | 0.030 | <0.0001 |
|  | Gender | 1.242 | 0.141 | <0.0001 |
|  | Age | 0.014 | 0.007 | 0.064 |
| Income | ADHD-PGS | -0.018 | 0.018 | 0.313 |
|  | Education | 0.133 | 0.008 | <0.0001 |
|  | Age | -0.019 | 0.002 | <0.0001 |

**Sex Differences**

We explored whether the relationship between the ADHD-PGS and GrimAge differed for men and women. We first constructed an OLS regression model to test for a statistical interaction. The OLS model was adjusted for age, gender, ancestral principal components, and included the interaction term between the ADHD-PGS and sex. There was no evidence for differences in the effects of the ADHD-PGS on GrimAge by sex (p=.13).

It should be noted that because the ADHD-PGS was derived from a large meta-analyses where effects of the genetic variants included from several samples were already adjusted for sex[5], and similarly the derivation of GrimAge also includes sex[6], there are limitations when estimating sex-specific differences in these relationships.

**References**

1. Okbay A, Beauchamp JP, Fontana MA, Lee JJ, Pers TH, Rietveld CA, Turley P, Chen G-B, Emilsson V, Meddens SFW: **Genome-wide association study identifies 74 loci associated with educational attainment**. *Nature* 2016, **533**(7604):539-542.

2. Ware E, Gard AM, Schmitz L, Faul J: **HRS Polygenic Scores – Release 4: 2006-2012 Genetic Data**. *Ann Arbor, Michigan: Survey Research Center, Institute for Social Research, University of Michigan* 2020.

3. Crimmins E, Kim J, Fisher J, Faul J: **HRS Epigenetic Clocks–Release 1**. *Survey Research Center, Univeristy of Michigan* 2020.

4. Horvath S: **DNA methylation age of human tissues and cell types**. *Genome biology* 2013, **14**(10):3156.

5. Demontis D, Walters RK, Martin J, Mattheisen M, Als TD, Agerbo E, Baldursson G, Belliveau R, Bybjerg-Grauholm J, Bækvad-Hansen M: **Discovery of the first genome-wide significant risk loci for attention deficit/hyperactivity disorder**. *Nat Genet* 2019, **51**(1):63-75.

6. Lu AT, Quach A, Wilson JG, Reiner AP, Aviv A, Raj K, Hou L, Baccarelli AA, Li Y, Stewart JD *et al*: **DNA methylation GrimAge strongly predicts lifespan and healthspan**. *Aging (Albany NY)* 2019, **11**(2):303-327.
